# Viscosity measurement of molten alumina and zirconia using aerodynamic levitation, laser heating and droplet oscillation techniques

**DOI:** 10.1016/j.heliyon.2023.e22424

**Published:** 2023-11-17

**Authors:** Yaopeng Gong, Li zhang, Yidan Yuan, Qiang Guo, Weimin Ma, Shanfang Huang

**Affiliations:** a Department of Engineering Physics, Tsinghua University, Beijing, China; b China Nuclear Power Engineering Co., Ltd (CNPE), Beijing, China; c Royal Institute of Technology (KTH), Stockholm, Sweden

**Keywords:** *Viscosity*, *Alumina*, *Zirconia*, *Aerodynamic levitation*, *Laser heating*, *Droplet oscillation*

## Abstract

Reliable thermophysical properties of core melt (corium) are essential for the accurate prediction of the severe accident progression in light water reactors. Zirconia is one of the most important materials in corium. Despite the high interest in the viscosity of molten zirconia, few experimental data have been reported due to its high melting temperature and high vapor pressure. In the present study, the viscosity of molten zirconia was measured using aerodynamic levitation, laser heating and droplet oscillation techniques. A material sample was levitated by argon gas flow in a conical nozzle and then melted into a droplet by laser beams. The initial quiescent droplet was forced to oscillate by the excitation of a loudspeaker, and the viscosity was deduced based on the characteristics of the droplet damped oscillation after the loudspeaker was turned off. The viscosity of molten alumina was first measured for verification of the measurement system. Afterwards the viscosity of molten zirconia was measured. The results showed that the viscosity of molten zirconia at melting temperature (2988K) was 12.87 ± 1.03 mPa s and decreased with increasing temperature. The measurement uncertainties are within 21 %.

## 1 Introduction

When a severe accident occurs in a light water reactor, the core materials will melt and form a mixture of UO_2_/ZrO_2_/Zr/Fe − so-called corium. The behavior of corium plays an important role in severe accident progression, and its prediction is crucial to the assessment of corium migration and coolability, debris formation and molten core concrete interaction [1]. For the prediction of corium behavior, models and computer codes have been developed to simulate various severe accident phenomena. In such models and simulation codes, thermophysical properties of corium are among the necessary input parameters. Therefore, thermophysical properties of corium are required to predict the flow behavior of corium.

The present study is motivated to measure the viscosity − one of the most important thermophysical properties. In particular, the focus is placed on zirconia (ZrO_2_), since it is an influential component of corium. For instance, the zirconia accounts for 13.75 % of the oxides in the corium of Three Mile Island accident [2,3]. Despite the high interest in the viscosity of molten zirconia, few experimental data have been reported due to its high melting temperature and high vapor pressure. Several data of zirconia viscosity have been estimated through the Molecular Dynamics (MD) method [4,5] with low reliability. Moreover, the viscosity of molten zirconia above its melting temperature was measured through aerodynamic levitation (ADL) [6,7]. However, the viscosity values from the experiment are significantly higher than those from the MD method. There is a clear need to have more experimental data on molten zirconia for verification and to support the study of corium behavior.

Viscosity at room temperature is easy to measure with sufficient accuracy using a proper technique. However, it is difficult to measure the viscosity of molten oxides due to low viscosities and high melting temperatures. Traditional contact methods for viscosity measurement at high temperatures include the ultrasonic sensor method [8] and the oscillation cup method (also named the rotating bob method) [[9], [10], [11], [12]]. The TIGEL facility [13] developed in Russia adopted the oscillation cup method to measure the corium viscosity including molten UO_2_–ZrO_2_ and UO_2_–ZrO_2_–Zr. The viscosity of SiO_2_–CaO–Al_2_O_3_ slag has also been measured through the oscillation cup method [14]. The vibrating Finger method is a new contact technique developed for viscosity measurement up to 1900K [15]. However, all contact methods are easily hampered by interactions between the sample and crucible.

To avoid sample-crucible interactions at high temperatures, contactless methods, such as electromagnetic levitation (EML) [[16], [17], [18]], electrostatic levitation (ESL) [19,20], acoustic levitation (AL) [21], gas-film levitation (GFL) [[22], [23], [24]], aerodynamic levitation [6,[25], [26], [27]] and hybrid levitation [28,29], are developed for thermophysical property measurements above 1800K. Among these contactless methods, EML and ESL are mainly applicable to metallic materials with good electric conductivity. The viscosity of Zr_*x*_O_1-*x*_ has been measured by ESL [30] since metal zirconium existed in the Zr–O binary system and the samples could be charged easily. For the AL method, a high-density sample requires greater acoustic intensity, causing strong droplet oscillation, atomization and fragmentation. The VITI facility developed in France adopted the GFL method to measure the viscosity of oxides including a nuclear glass [31]. However, it is difficult for VITI to heat samples above 3000K using electromagnetic induction heating. For the hybrid levitation, the levitation stability becomes much better but the devices are much more complex and costs are much higher. The ADL method is the most appropriate technique to levitate oxides such as alumina and zirconia. In addition to the ability to measure density, surface tension and viscosity, the ADL technique also has strong scalability advantages and can be used for the measurements of crystal structure [32], melting temperature and heat capacity [33,34]. The viscosity of alumina has been measured through ADL [6,25,26] by many researchers and the results are in good agreement. However, only a few published papers have presented the viscosity measurement of molten zirconia through aerodynamic levitation [6,7]. More data are needed for verification and comparison.

This paper first introduces an experimental setup designed to measure the thermophysical properties of corium components. Preparation, levitation and damped oscillation of alumina and zirconia samples are then presented. Finally, based on the measured signals and data analysis, the viscosity values of molten alumina and zirconia are obtained. The viscosity of molten alumina is used to verify the measurement system through a comparison with known data.

## 2 Experimental setup

**Fig. 1:**
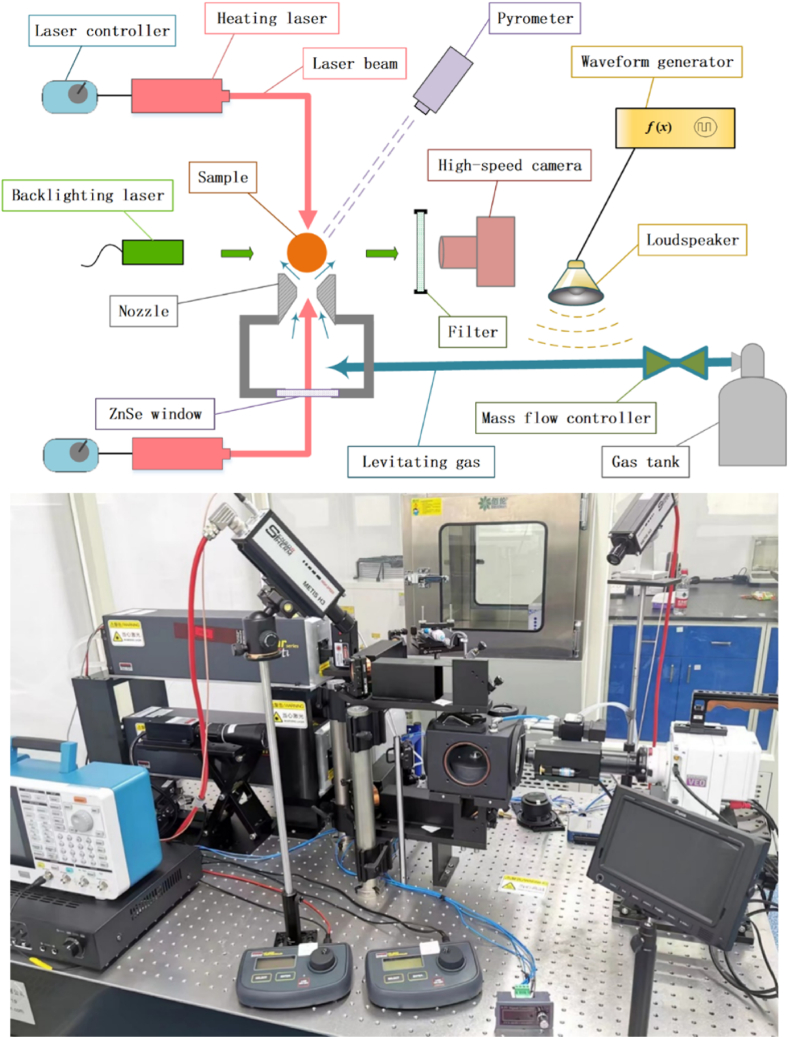
Schematic view and picture of ALSEE facility.

The experimental setup, named ALSEE (Aerodynamic Levitation-laSEr hEating installation for melt properties), is illustrated in Fig. 1. It is designed for thermophysical property measurements of molten corium components (UO_2_/ZrO_2_/Zr/Fe and their mixtures), including density, surface tension and viscosity. The conceptualization of this facility draws inspiration from the design conceptualized by Langstaff et al. [26]. Notably, their pioneering work involving the utilization of acoustic excitation significantly facilitates the measurements of surface tension and viscosity at elevated temperatures. The density of alumina and zirconia has been measured in ALSEE [35,36]. The focus of this paper lies in the viscosity measurements pertaining to molten alumina and zirconia.

### 2.1 Levitation and heating

The argon gas stored in the gas tank was depressurized to approximately 200 KPa before entering the conical nozzle. Samples with diameters of 2–3 mm were levitated above the nozzle. To ensure optimal levitation stability, the argon gas flow rate was finely regulated using a mass flow controller (KOFLOC EX250SC).

Two 100 W CO_2_ laser devices (Synrad ti-100HS) were placed up and down to heat the upper and lower sides of the sample (see Fig. 1). The laser beams were directed towards the sample using flat mirrors and focusing lenses. Each laser emitted a 10.6 μm wavelength beam with an initial diameter of 2 mm. After passing through the focusing lens, the beam diameter reduced to approximately 1 mm at the sample surface. High stability of the laser output power (2 %) was crucial to ensure precise temperature control.

The sample temperature was monitored using a pair of bichromatic pyrometers (Sensortherm H311). The first pyrometer exhibited a measurement range from 1173K to 2073K, with wavelengths for Channel 1 falling within 0.93–1.1 μm and for Channel 2 within 0.75–0.93 μm. Correspondingly, the second pyrometer exhibited a measurement range from 1873K to 3573K, with wavelengths for Channel 1 at 0.99 μm and for Channel 2 at 0.78 μm. Both pyrometers had a measurement uncertainty of 0.5 %, a critical feature ensuring accurate temperature readings.

In essence, a bichromatic pyrometer operates based on a two-color infrared measurement principle. This methodology involves assessing the ratio of spectral radiant brightness from the target object at two selected wavelengths. It's important to emphasize that this technique capitalizes on the assumption of equivalent object emissivities at the chosen wavelengths. As a consequence, the measured temperature becomes solely contingent on the ratio and remains independent of the emissivity. This intrinsic characteristic eliminates the need for temperature corrections, which is a significant advantage in utilizing bichromatic pyrometers for temperature measurements in scenarios involving materials with elusive emissivity information.

### 2.2 Image processing

**Fig. 2:**
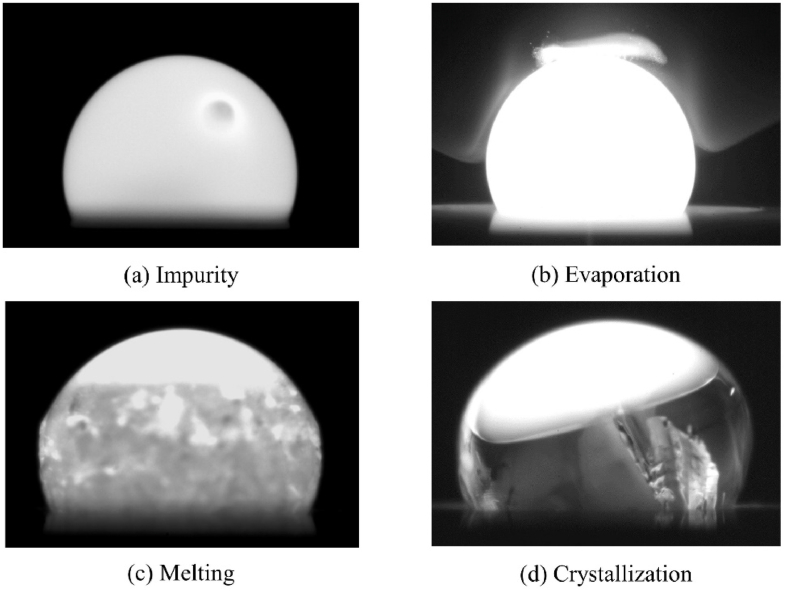
Observations by self-illumination imaging. (a) Impurity. (b) Evaporation. (c) Melting. (d) Crystallization.

The contour of the samples was captured using a high-speed camera (Phantom VEO440L) equipped with a zoom lens (Movetem MAZ12.0 × LZ). Throughout the measurement, the camera was set up with a resolution of 896 × 600 pixels. To achieve sharp edges, shadow photography could be employed by utilizing a backlighting laser and a narrow-band filter. However, the experimental setup opted for a conventional photography technique, specifically self-illumination imaging. This technique was chosen as a means to directly capture the characteristics of the samples when subjected to elevated temperatures. Notably, this approach offers the advantage of enabling real-time observations of many phenomena, including the visual tracking of impurity presence (Fig. 2a), alongside the dynamic processes of sample evaporation (Fig. 2b), melting (Fig. 2c), and crystallization (Fig. 2d).

To convert the sample diameters from pixels to standard length units for viscosity measurement, a calibration process was conducted. Standard stainless-steel spheres with diameters ranging from 2.0 mm to 2.5 mm were used. The standard spheres were loaded on a conical nozzle and photographed by the high-speed camera. By comparing the known accurate diameters of the standard spheres with their corresponding pixel measurements, calibration results were obtained as presented in Table 1. It turned out that the length of one pixel corresponded to 5.166 μm.

**Table 1:** High-speed camera calibration.

Material	Diameter (mm)	Diameter in image (pixel)	Camera calibration (μm/pixel)
Stainless-steel	2.0	387.52	5.161
Stainless-steel	2.1	405.68	5.176
Stainless-steel	2.2	426.36	5.160
Stainless-steel	2.3	445.26	5.166
Stainless-steel	2.4	464.96	5.162
Stainless-steel	2.5	483.48	5.171
Average			5.166

The sample surface profile was determined using the Canny edge detection technique, known for its superior performance compared to other techniques such as Sobel, Robert, Prewitt, Laplacian, and Laplace of Gaussian [37]. After the sample melted above the nozzle, it exhibited an ellipsoidal shape due to the joint effect of gravity and argon gas shear force. In a previous study, the least-squares fitting based on algebraic distances (LSA) using the pseudo-inverse technique was used for the density measurement of molten alumina [35]. However, the LSA algorithm was found to be susceptible to data noise and outliers due to its assumption of zero mean noise [38]. To mitigate the impact of noise and outliers, a robust algorithm called M-estimator (MEST) was utilized for ellipse fitting in the present study.

The MEST algorithm is an iterative method that builds upon the LSA algorithm. The 2-D elliptic equation can be described in Eq. (1):

(1) $${Q\left(x,y\right)=Ax}^{2}+2Bxy+Cy^{2}+2Dx+2Ey+F=0$$

Under the normalization of *A + C* = 1, the ellipse can be described by a vector p as shown in Eq. (2):

(2) $$p={\left[A,B,D,E,F\right]}^{T}$$

A common approach for ellipse fitting is to minimize the algebraic distance with given points $\left(x_{i},y_{i}\right)$. This involves minimizing the function defined in Eq. (3):

(3) $$\Psi \left(p\right)=\sum_{i=1}^{n}Q^{2}\left(x_{i},y_{i}\right)$$

The LSA algorithm using the pseudo-inverse technique provides a solution for the vector p by disregarding the influence of data noise as shown in Eq. (4):

(4) $$p={\left(A^{T}A\right)}^{-1}A^{T}b$$

where A and b are matrices constructed from the coordinates of the given points, as described in Gong et al. [35]. The MEST algorithm aims to mitigate the influence of noise and outliers by replacing the squared residuals $Q^{2}\left(x_{i},y_{i}\right)$ with another function of the residuals in Eq. (5):

(5) $$\Psi \left(p\right)=\sum_{i=1}^{n}f\left(Q\right)$$

where f is a symmetric, positive-definite function with a unique minimum at zero, chosen to be less sensitive to outliers than the square function. In the present study, the Cauchy function is employed as the replacement function, which is defined in Eq. (6):

(6) $$f\left(x\right)=\frac{1}{2}\log\left(1+x^{2}\right)$$

Taking the partial derivative of Eq. (5) with respect to the vector p and setting it to zero result in Eq. (7):

(7) $$\sum_{i=1}^{n}\frac{\partial f\left(Q\right)}{\partial Q}\frac{\partial Q}{\partial p}=0$$

The elliptical parameters A,B,C,D,E,F are obtained by solving Eq. (7). In accordance with the Cauchy function in Eq. (6), the influence and weight functions are defined in Eqs. (8), (9), respectively:

(8) $$\psi \left(x\right)=\frac{\partial f\left(x\right)}{\partial x}=\frac{x}{1+x^{2}}$$

(9) $$\omega \left(x\right)=\frac{\partial \psi \left(x\right)}{\partial x}=\frac{1}{1+x^{2}}$$

where ψ(x) is called the influence function and ω(x) the weight function. Eq. (7) now can be expressed as Eq. (10):

(10) $$\sum_{i=1}^{n}\omega \left(Q\right)Q\frac{\partial Q}{\partial p}=0$$

Eq. (10) is exactly the equation to solve the iterated reweighted least-squares problem as shown in Eq. (11):

(11) $$\Psi =\sum_{i=1}^{n}{\omega \left(Q^{k-1}\right)Q}^{2}\left(x_{i},y_{i}\right)$$

The algorithm for solving Eq. (11) is identical to the LSA algorithm. Once the elliptical parameters A,B,C,D,E,F are obtained, the semi-major and semi-minor axes can be determined by Eqs. (12), (13), respectively:

(12) $$r_{a}=\sqrt{\frac{2\left(AE^{2}+CD^{2}+FB^{2}-2BDE-ACF\right)}{\left(B^{2}-AC\right)\left[\sqrt{{\left(A-C\right)}^{2}+4B^{2}}-\left(A+C\right)\right]}}$$

(13) $$r_{b}=\sqrt{\frac{2\left(AE^{2}+CD^{2}+FB^{2}-2BDE-ACF\right)}{\left(B^{2}-AC\right)[-\sqrt{{\left(A-C\right)}^{2}+4B^{2}}-\left(A+C\right)]}}$$

If *A* < *C*, the ellipse inclination angle can be determined by Eq. (14):

(14) $$\theta =\frac{1}{2}\cot^{-1}\left(\frac{A-C}{2B}\right)$$

If *A* > *C*, the ellipse inclination angle can be determined by Eq. (15):

(15) $$\theta =\frac{\pi }{2}+\frac{1}{2}\cot^{-1}\left(\frac{A-C}{2B}\right)$$

To verify the robustness of the MEST algorithm, a comparison involving the fitting of an ellipse using three different algorithms is presented. The initial step involves defining the parameters of the ellipse. The coordinates of the ellipse center are set as (120,100), while the semi-major and semi-minor axis are 250 and 100 respectively, and the inclination angle is 45° (0.7854 in radian). Subsequently, a dataset comprising 300 points extracted from the ellipse is generated. To simulate realistic conditions, random number generation is employed to introduce noise to the point coordinates. Further complexity is incorporated by introducing 30 outliers, culminating in a composite dataset characterized by noise and outliers. This dataset serves as the foundation for ellipse fitting conducted through three algorithms: LSA, GWLS and MEST. It is worth noting that the GWLS algorithm is a gradient-weighted least-squares fitting algorithm, and the details can be found in Ref. [38].

**Fig. 3:**
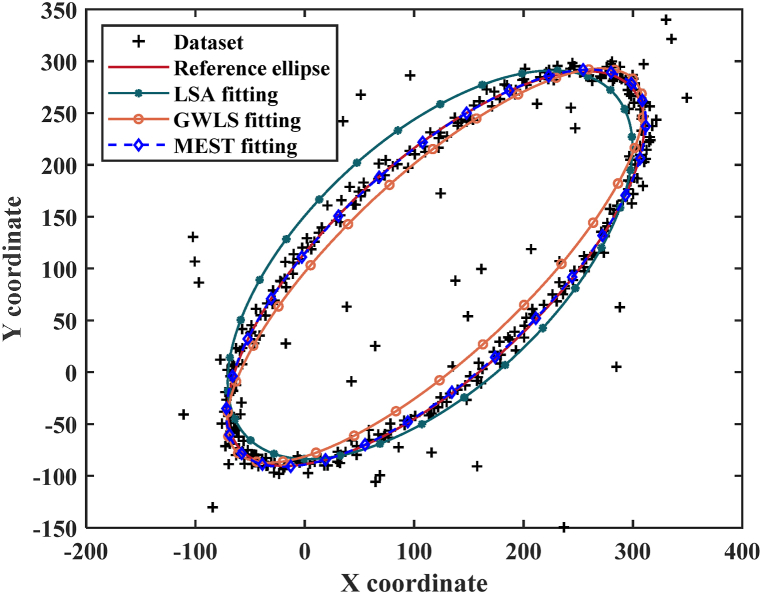
Ellipse fitting by LSA, GWLS and MEST algorithms.

Visual examination in Fig. 3 offers an immediate insight into the outcomes of these algorithms. The ellipses obtained from the LSA and GWLS algorithms notably deviate from the reference ellipse, indicating their limited robustness in the presence of noise and outliers. This observation aligns with the analysis in Table 2, where the fitted values derived from the MEST algorithm exhibit remarkable proximity to the reference values. Notably, the maximum deviation stands at a mere 0.39 %. This underlines the profound efficacy of the MEST algorithm in mitigating the influence of both noise and outliers, thereby significantly enhancing the precision of semi-major and semi-minor axis determinations. The exceptional adaptability of the MEST algorithm becomes particularly pronounced in scenarios marked by localized abnormal deformations on droplet surfaces or the emergence of noise in image backgrounds.

**Table 2:** Robustness verification of the MEST algorithm.

Ellipse parameter	Reference value	Fitted value and deviation		
		LSA	GWLS	MEST
X coordinate of center	120	114.44 (4.63 %)	118.88 (0.93 %)	119.95 (0.04 %)
Y coordinate of center	100	103.41 (3.41 %)	102.46 (2.46 %)	99.96 (0.04 %)
Semi-major axis	250	233.99 (6.40 %)	253.39 (1.36 %)	249.17 (0.33 %)
Semi-minor axis	100	117.67 (17.67 %)	85.20 (14.80 %)	100.25 (0.25 %)
Inclination angle	0.7854	0.7987 (1.69 %)	0.7875 (0.27 %)	0.7885 (0.39 %)
Average of deviation		6.76 %	3.96 %	0.21 %

### 2.3 Damped oscillation

Viscosity was measured by introducing an initial deformation to the levitated droplet which lead to a damped oscillation. The measurement technique employed on the VITI facility was based on the relaxation kinetics of a deformed droplet [22]. To induce the deformation, a hydraulic jack was connected to the top porous membrane, allowing for a sudden elevation of the droplet. In the case of a low-viscosity droplet, the return of the droplet apex to equilibrium took the form of a damped oscillation. However, due to significant deviations in the droplet shape from a perfect sphere, correction factors were used, resulting in larger viscosity uncertainty.

To overcome these challenges, the acoustic excitation technique developed by Langstaff et al. [26] was used in the present study. This technique involved forcing the droplet to oscillate in a predictable way using acoustic waves, where the oscillation frequencies precisely matched the acoustic wave frequencies. A frequency generator equipped with an amplifier was used to generate a sinusoidal wave voltage. This voltage was then used to drive a loudspeaker, which excited the droplet to oscillate in the *l* = 2, *m* = 0 mode. This oscillation mode exhibited axisymmetry, with the axis of symmetry aligned vertically [39]. After the loudspeaker was turned off, the droplet radius underwent a damped oscillation.

### 2.4 Viscosity measurement

Image processing based on the MEST algorithm was conducted frame by frame to extract the center coordinates as well as the semi-major and semi-minor axes of the droplet. However, it should be noted that during the droplet oscillations, the directions of the semi-major and semi-minor axes may be reversed. In order to determine the horizontal radius of the droplet, the inclination angle needs to be taken into account. Consequently, the time evolution of the horizontal radius takes the form of a damped oscillation as shown in Eq. (16):

(16) $$r_{h}\left(t\right)=A∙\exp\left(-\Gamma t\right)∙\sin\left(2\pi \nu t+\varphi \right)+r_{av}$$

where A is the amplitude, Γ the damping constant, ν the excitation frequency, φ the phase and $r_{av}$ the mean radius at rest.

To obtain the damping constant, a nonlinear least squares fitting approach is employed. By fitting the observed data to the damped oscillation model, the damping constant can be estimated. Once the damping constant was determined, the viscosity can be derived through Eq. (17):

(17) $$\eta =\frac{\rho R^{2}}{5}\Gamma$$

where ρ is the density and R the equivalent radius of the droplet. If the density and mass of the droplet are known, the equivalent radius can be determined in Eq. (18):

(18) $$R=\sqrt[{3}]{\frac{3m}{4\pi \rho }}$$

where m is the sample mass. Then, the viscosity can also be calculated in Eq. (19):

(19) $$\eta =\frac{1}{5}{\left(\frac{3}{4\pi }\right)}^{\frac{2}{3}}m^{\frac{2}{3}}\rho^{\frac{1}{3}}\Gamma$$

According to the propagation law, the uncertainty of viscosity can be determined by considering the uncertainties in the measured parameters including mass, density and damping constant as shown in Eq. (20):

(20) $$\frac{\Delta \eta }{\eta }=\sqrt{\frac{4}{9}{\left(\frac{\Delta m}{m}\right)}^{2}+\frac{1}{9}{\left(\frac{\Delta \rho }{\rho }\right)}^{2}+{\left(\frac{\Delta \Gamma }{\Gamma }\right)}^{2}}$$

where Δ means the uncertainty of a variable.

## 3 Results and discussion

### 3.1 Sample preparation and levitation

**Fig. 4:**
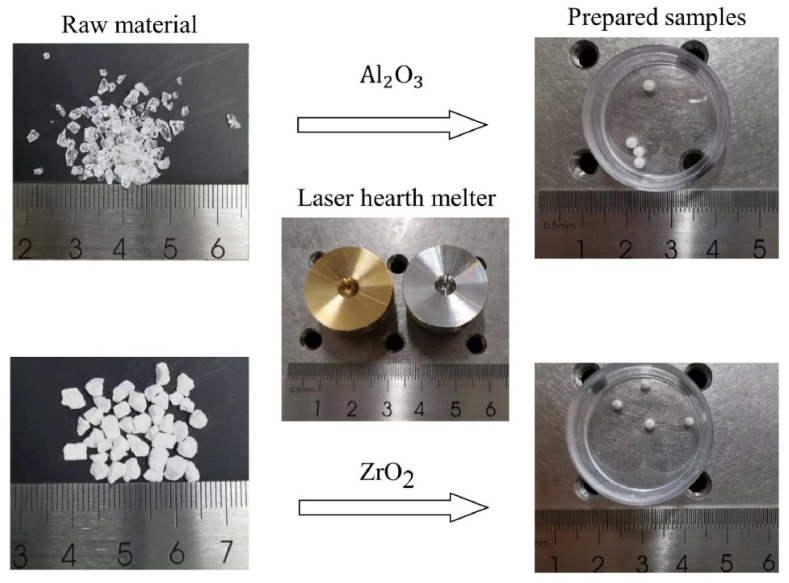
Samples of alumina and zirconia prepared in the laser hearth melter.

In the present study, the alumina raw materials were in the form of small crystals, whereas zirconia raw materials were small pieces that were pressed from powders. The samples were prepared using a laser hearth melter device, as shown in Fig. 4. The raw materials were melted using laser heating, resulting in the formation of spherical samples with diameters ranging from 2 to 3 mm. Both the alumina and zirconia samples had a white color, but the alumina samples appeared more translucent compared to the zirconia samples. The purity of the raw materials was more than 99.9 %. The induced impurities were negligible for the samples prepared using the laser hearth melter [40]. Therefore, samples contained few impurities and the effect on viscosity measurement could also be negligible.

**Fig. 5:**
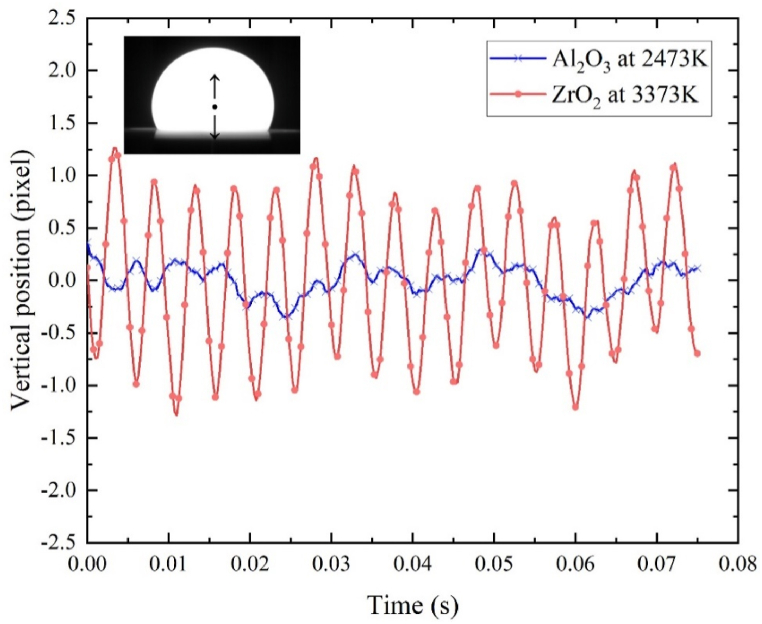
Sample fluctuation upward and downward the equilibrium position.

A sample with a diameter of 2–3 mm was levitated above the conical nozzle using a gas flow rate of approximately 0.4 L/min. By applying laser heating, the sample underwent a phase change and transformed into a molten droplet. It is worth noting that the lower part of the droplet was hidden by the conical nozzle as depicted in Fig. 5. During the experiment, the droplet fluctuated around its equilibrium position. The vertical position of the droplet was determined by image processing of the droplet visible portion. The resulting changes in the vertical position of the molten alumina and zirconia droplets, measured in pixels, are also presented in Fig. 5.

Based on the calibration of 5.166 μm/pixel, the observed vertical fluctuation of the molten alumina droplet is approximately 5 μm at 2473K, while that of the molten zirconia droplet is around 15 μm at 3373K. By optimizing the structure of the nozzle, the levitation stability in the present study is improved when compared to an ESL experiment [41] with a fluctuation of 100 μm and an ADL experiment [42] with a fluctuation of 40 μm. The larger fluctuation observed in the molten zirconia droplet compared to molten alumina can be attributed to several factors. Firstly, zirconia has a higher melting temperature, which leads to a higher temperature gradient in the flow field. This increased temperature gradient contributes to lower flow stability. Additionally, zirconia evaporates faster than alumina above their melting temperatures. The evaporated zirconia can then solidify at the low-temperature nozzle wall, disrupting the symmetry of the argon gas channel and weakening the levitation stability.

### 3.2 Damped oscillation

During the stable levitation of a sample droplet, a sine voltage signal was applied to the loudspeaker which served to excite the droplet and induced oscillation specifically in the *l* = 2, *m* = 0 mode. After a few seconds of oscillation, the loudspeaker was turned off, and the droplet commenced a damped oscillation. This damped oscillation continued until the droplet eventually reached a steady-state condition. To capture and monitor the damped oscillation process, the high-speed camera was utilized, operating at a frame rate of 2000 fps with a resolution of 896 × 600 pixels. After the loudspeaker was turned off, the horizontal radius of the droplet continued to oscillate due to inertia but gradually decayed over time due to the presence of viscosity. The rate of decay is determined by the sample density, viscosity and size. In the case of oxide droplets like alumina and zirconia, which typically have viscosities ranging from 5 to 50 mPa s, and a diameter of 2.5 mm, the decay time (means the reciprocal of damping constant) of the oscillation is estimated to be around 0.02–0.5 s.

**Fig. 6:**
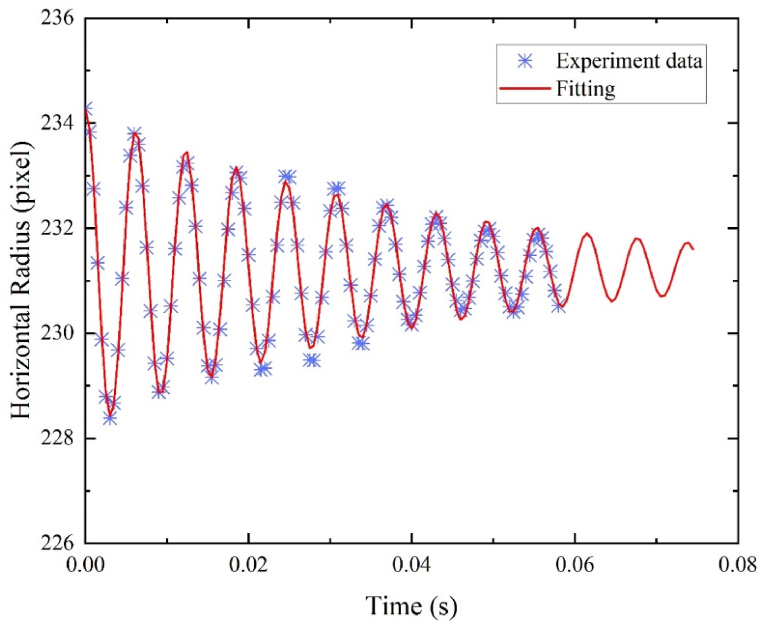
Damped oscillation of molten alumina at 2571K.

Fig. 6 illustrates the variation of the horizontal radius in pixels during the damped oscillation of an alumina droplet at a temperature of 2571K. The experimental observations confirm the occurrence of the damped oscillation following the cessation of acoustic excitation. The fitting results of the horizontal radius data are presented in Table 3, along with the fitting coefficients and their 95 % confidence bounds. The damping constant for molten alumina at 2571K is determined to be 24.52 s^−1^, which will be used in the subsequent viscosity calculations. Considering the camera calibration result of 5.166 μm/pixel, the mean radius at rest of 231.22 pixels corresponds to a radius of 1.19 mm. The fitting quality of the data is assessed through various metrics. The Sum of Square Error (SSE) is found to be 2.5839, indicating a small discrepancy between the fitted curve and the actual data points. The Root Mean Square Error (RMSE) is 0.1592, further confirming the accuracy of the fitting. The Degrees of Freedom Adjusted R-square (DFE) is defined as the difference between the number of response values and the number of fitted coefficients. The obtained R-square value of 0.9860 and adjusted R-square value of 0.9855, both of which are close to 1, indicate a high degree of fitting satisfaction.

**Table 3:** Fitting results for molten alumina at 2571K

Parameter		Fitting goodness	
A	3.078 (2.963, 3.192)	SSE	2.5839
Γ	24.52 (22.75, 26.28)	R-square	0.9860
ν	162.7 (162.4, 163.0)	DFE	102
φ	1.028 (0.9887, 1.067)	Adjusted R-square	0.9855
$r_{av}$	231.22 (213.18, 213.25)	RMSE	0.1592

High-quality data is crucial for achieving accurate and reliable fitting results. In the case of levitation instability, the data quality can be compromised as the droplet undergoes fluctuations and deformations, making the fitting process more challenging. Furthermore, asymmetry or rotation of the levitated droplet, possibly caused by the perturbation of the flowing argon gas, can further impact the accuracy of the fitting. In such cases, the observed horizontal radius may drift over time. It is worth noting that even with lower quality data, it is still possible to obtain fitting coefficients. However, the uncertainties associated with these coefficients are likely to be higher, reflecting the reduced confidence in the accuracy of the fitting results.

### 3.3 Viscosity of molten alumina

As shown in Table 3, the damping constant for molten alumina at 2571K was determined to be 24.52 s^−1^. The density was 2871 kg/m^3^ at 2571K according to Eq. (21) from Gong et al. [35]:

(21) $$\rho =-0.2421\times T+3493\left(\text{kg}/m^{3}\right)$$

**Fig. 7:**
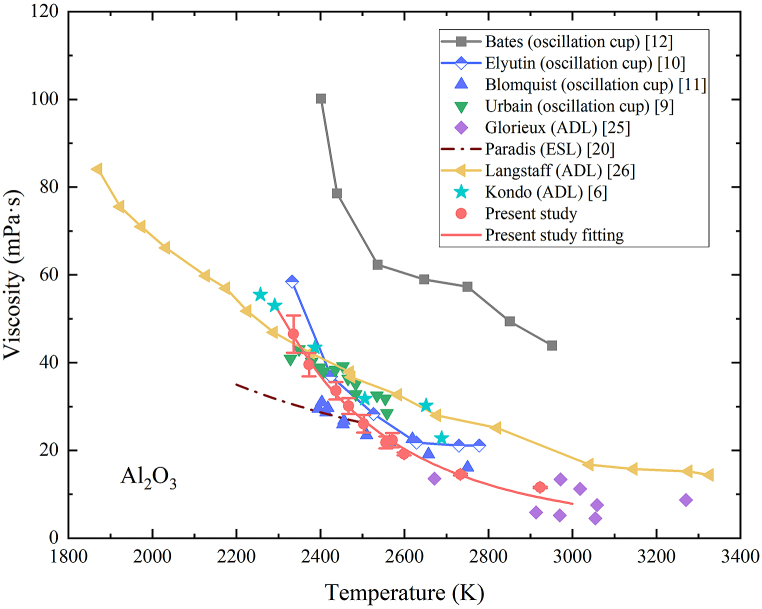
Temperature-dependent viscosity of molten alumina.

Considering the sample mass of 23.98 mg, the viscosity at 2571K was 22.30 mPa s according to Eq. (19). Fig. 7 shows the temperature-dependent viscosity of molten alumina measured in the present study, along with published data obtained using the oscillation cup [[9], [10], [11], [12]], ESL [20] and ADL [6,25,26] methods.

According to Eq. (20), the uncertainty of viscosity can be calculated by considering the uncertainties associated with mass, damping constant, and density. The uncertainty of mass is a composite outcome of the mass loss resulting from evaporation and the measurement accuracy of the employed scale as shown in Eq. (22):

(22) $$\Delta m=\sqrt{{\left(\Delta m_{e}\right)}^{2}+{\left(\Delta m_{s}\right)}^{2}}$$

where $\Delta m_{e}$ is the mass loss due to evaporation and $\Delta m_{s}$ the uncertainty of the employed scale which is 0.05 mg. Viscosity data of alumina at different temperatures were measured from only one sample. The measurement took about 20 min to obtain good levitation stability at different temperatures. The mass before and after the levitation was 20.98 mg and 20.73 mg respectively. Therefore, the mass loss was 0.25 mg and the uncertainty of mass was then determinded to be 0.255 mg. It is noted that the mass uncertainty is mainly determind by evaporation since the mass loss resulting from evaporation is significantly greater than the scale uncertainty in most cases. The density uncertainty, as mentioned in Gong et al.’s work [35], is reported to be 1.40 %. The damping constant uncertainty is determined through the damped oscillation fitting process. The maximum uncertainty of viscosity is 9 % at 2336K for molten alumina. As shown in Fig. 7, the uncertainty associated with viscosity exhibits a downward trend as the temperature increases. This is because as the viscosity of the alumina increases, the oscillation of the droplet experiences a more rapid decay, resulting in a shorter time interval available for data collection. Consequently, there are fewer data available for the fitting process, leading to higher uncertainty of the damping constant. This elevated uncertainty of the damping constant further increases the uncertainty of the viscosity

The literature values for viscosity of molten alumina at high temperatures exhibit significant variations, primarily due to the inherent challenges in accurately measuring viscosity at high temperatures. Bates et al. [12] reported viscosity values that deviate significantly from other reported data, possibly due to the potential interactions between the sample and crucible at high temperatures, which can affect the measurement accuracy. Glorieux et al. [25] reported a relatively high uncertainty of approximately 50 % since the damping constant was approximated from the width of the oscillation peak. Data values in Paradis et al. [20] are lower than others around the melting temperature, but is close to data in Bloomquist et al. [11] between 2400K–2500K. Advancements in techniques such as ADL and acoustic excitation have led to improved accuracy, as demonstrated by works of Langstaff et al. [26] and Kondo et al. [6]. The maximum difference between data of Langstaff et al. [26] and Kondo et al. [6] is about 18 %.

The viscosity values in the present study show good agreement with data reported by Blomquist et al. [11], with the maximum deviation of about 20 %. Good agreement is also shown with data reported by Glorieux et al. [25] taking account of associated uncertainty of around 50 %. The viscosity values in the present study demonstrate deviations below 6 % around melting temperature with the measurements conducted by Langstaff et al. [26] and below 20 % from 2336K to 2500K with the measurements conducted by Kondo et al. [6]. It is notable that measurements by Langstaff et al. [26] and Kondo et al. [6] utilized the same methodology as the present study. Within the temperature range of 2336K–2700K, our measured data occupies an intermediary position among published data. Different from other literature data, viscosity values in the present study exhibit a rapid downward trend as the temperature rises. This phenomenon might be interconnected with the rotation speed of the droplets. After the improvement of nozzle structure in the present study, the rotation speed is around 1 Hz which is smaller than other levitation expeiments [39]. Overall, the utilization of the ALSEE facility has proven effective in yielding accurate viscosity values for molten alumina within the temperature range of 2336K–2923K.

The viscosity of molten alumina decreases with increasing temperature. This behavior is contrary to the viscosity trend observed in gases, which typically increases in viscosity with rising temperature. The difference arises from the distinct intermolecular forces present in gases and liquids. In gases, higher temperatures lead to intensified molecular motion and enhanced momentum exchange, resulting in increased shear stress and viscosity. However, in liquids, higher temperature means the average distance between molecules will increase. As a result, the intermolecular gravitational force weakens, leading to a decrease in viscosity. The temperature-dependent viscosity of liquids can be described by the Andrade equation [43], which is shown in Eq. (23):

(23) $$\eta =\eta_{0}\;\exp\left[\frac{E_{a}}{R_{g}}\left(\frac{1}{T}-\frac{1}{T_{m}}\right)\right]$$

where $\eta_{0}$ is the viscosity at the melting temperature, $E_{a}$ the activation energy for viscous flow, $R_{g}$ the gas constant (8.314 J/(mol·K)) and $T_{m}$ the melting temperature. The viscosity data of molten alumina obtained in the present study are well fitted by the Andrade equation within the temperature range of 2336K–2923K in Eq. (24):

(24) $$\eta_{{\text{Al}}_{2}O_{3}}=\left(47.06\pm 2.15\right)\exp\left[\frac{(1.546\pm 0.144)\times 10^{5}}{R_{g}}\left(\frac{1}{T}-\frac{1}{2327}\right)\right]\left(\text{mPa}∙s\right)$$

The viscosity of alumina at its melting temperature (2327K) was determined to be 47.06 ± 2.15 mPa s. Additionally, the activation energy was found to be (1.546 ± 0.144) × 10^5^ J/mol. These values provide important insights into the flow behavior, fluidity and molecular interactions within molten alumina at high temperatures.

### 3.4 Viscosity of molten zirconia

The viscosity of molten zirconia was measured using the same method for alumina in the present study. The density of molten zirconia can be calculated by Eq. (25) according to the work of Zhang et al. [36]:

(25) $$\rho =-0.7202\times T+6869\left(\text{kg}/m^{3}\right)$$

**Fig. 8:**
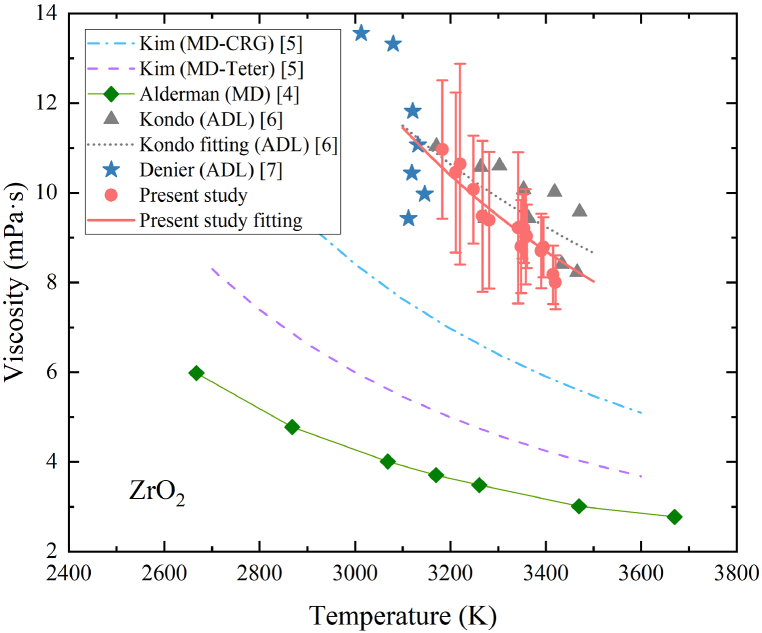
Temperature-dependent viscosity of molten zirconia.

Fig. 8 presents the temperature-dependent viscosity of molten zirconia in the temperature range of 3183K–3420K, along with data calculated by Kim et al. [5] and Alderman et al. [4] using the MD method and data measured by Kondo et al. [6] and Denier et al. [7] using the ADL method.

Viscosity data of zirconia at different temperatures were also measured from only one sample. The measurement only took about 2 min to reduce the effect of evaporation since molten zirconia evaporated significantly. The mass before and after the levitation was 20.33 mg and 19.86 mg respectively. Therefore, the mass loss was 0.47 mg and the uncertainty of mass was then determinded to be 0.473 mg. The density uncertainty, as mentioned in Zhang et al.’s work [36], is reported to be 2.33 %. The damping constant uncertainty is determined through the damped oscillation fitting process. The uncertainty associated with zirconia viscosity also exhibits a downward trend as the temperature increases for the same reason as alumina. The uncertainty of molten zirconia is greater than that of alumina. This can be attributed to several factors. Firstly, the levitation stability of molten zirconia is lower, resulting in a less symmetrical droplet compared to alumina, which adversely affects the quality of the obtained data. The lower quality data will lead to greater uncertainty in the damping constant. Secondly, the higher vapor pressure of zirconia results in a greater evaporation rate, introducing larger uncertainties in mass and density measurements. Ultimately, the maximum uncertainty is 21 % at 3220K in viscosity measurement for molten zirconia, indicating the challenges associated with accurately measuring the viscosity of molten zirconia.

Viscosity values calculated by Alderman et al. [4] are notably lower compared to other values. This discrepancy can be attributed to estimations of parameters in the Stokes-Einstein equation which is not accurate. Kim et al. [5] employed two potential models, CRG and Teter, to calculate the activation energy required in the predictive Andrade model. However, there exists a certain level of discrepancy between the CRG and Teter models, indicating the sensitivity of MD calculations to the choice of models. Therefore, the accuracy of MD calculations is challenging to guarantee. In contrast, viscosity values obtained using the ADL method in the present study demonstrate reasonable agreement with the values experimentally measured by Kondo et al. [6] and Denier et al. [7], taking into account the uncertainties associated with viscosity. Data recently measured by Denier et al. [7] exhibit a larger scatter in a narrow temperature range. The viscosity may be also slightly overestimated due to the multimode oscillation. It will be more difficult to obtain an accurate fitted equation from viscosity data with larger fluctuations. The quality of zirconia viscosity data measured in the present study is better and the viscosity data are fitted by the Andrade equation within the temperature range of 3183K–3420K in Eq. (26):

(26) $$\eta_{{\text{ZrO}}_{2}}=\left(12.87\pm 1.03\right)\exp\left[\frac{\left(8.035\pm 2.010\right)\times 10^{4}}{R_{g}}\left(\left(\frac{1}{T}-\frac{1}{2988}\right)\right)\right]\left(\text{mPa}∙s\right)$$

The viscosity of zirconia at its melting temperature (2988K) was then determined to be 12.87 ± 1.03 mPa s. Additionally, the activation energy was found to be (8.035 ± 2.010) × 10^4^ J/mol. The fluctuation in zirconia viscosity is slightly reduced compared with that in Kondo et al. [6]. Therefore, the uncertainty of the coefficients in the Andrade equation is smaller in the present study. However, it's crucial to underscore that despite this enhanced precision, the activation energy remains within a notable level of uncertainty. For the realization of a more precise activation energy estimation, it becomes imperative to dedicate further endeavors to refining experimental techniques. A central objective is the improvement of molten zirconia's levitation stability throughout the viscosity measurement process.

**Fig. 9:**
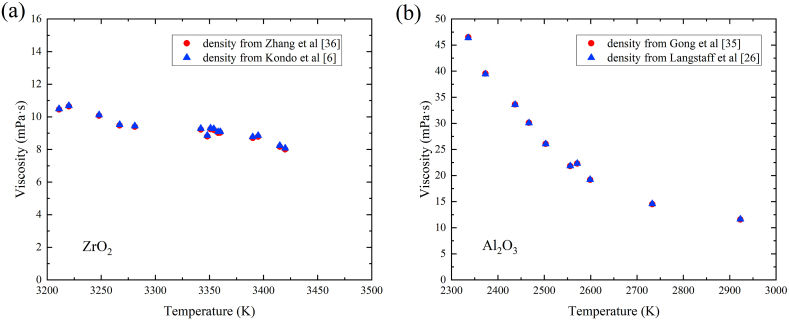
viscosity recalculated using different density sources. (a) Zirconia. (b) Alumina.

A prominent concern associated with the utilization of the self-illumination imaging method is the potential for overestimating the sample radius, thereby introducing errors in viscosity determinations. In the present study, viscosity is related to sample mass, density and damping constant as depicted in Eq. (19). Notably, the imaging methodology has no bearing on the determination of mass. The overestimation of radius yields a subtle impact on the determination of the damping constant since the damping constant is deduced from the damping process, and the overestimation mainly contributes to the determination of mean radius at rest in Eq. (16). To assess the influence of imaging methods, specifically self-illumination and backlighting imaging, on viscosity determination, recalculations are conducted. Alumina density data from Langstaff et al. [26] and zirconia density data from Kondo et al. [6] are employed for evaluation. The recalculated viscosity values are then juxtaposed for comparison in Fig. 9a and b. Moreover, the viscosity data for molten alumina and zirconia undergo Andrade equation fittings again, yielding outcomes outlined in Table 4 and Table 5.

**Table 4:** Andrade equation coefficients of alumina viscosity.

Andrade equation Coefficient	Using density from self-illumination in Gong et al. [35]	Using density from backlighting in Langstaff et al. [26]	Deviation
$\eta_{0}$	47.06 ± 2.15	46.89 ± 2.16	0.36 %
$E_{a}$	$\left(1.546\pm 0.144\right)\times 10^{5}$	$\left(1.536\pm 0.145\right)\times 10^{5}$	0.65 %

**Table 5:** Andrade equation coefficients of zirconia viscosity.

Andrade equation Coefficient	Using density from self-illumination in Zhang et al. [36]	Using density from backlighting in Kondo et al. [6]	Deviation
$\eta_{0}$	12.87 ± 1.03	12.94 ± 1.00	0.54 %
$E_{a}$	$\left(8.035\pm 2.010\right)\times 10^{4}$	$\left(8.032\pm 1.940\right)\times 10^{4}$	0.04 %

Despite the different temperature coefficients apparent in the density measured from self-illumination and backlighting methods, the differences in density values are inconsequential and the disparities in viscosity exhibit minimal magnitudes as shown in Fig. 9a and b. The deviations in coefficients shown in tables 4 and 5 remain below 0.65 %. Therefore, a conclusion can be drawn that varying density data from different imaging methods exert a negligible impact on viscosity determinations.

Attention should be paid to the evaporation phenomenon during the viscosity measurement of molten zirconia. The vapor pressure of zirconia, as calculated by Kondo et al. [6], was found to be 18 Pa, whereas for alumina it was 0.12 Pa. This significant difference in vapor pressure highlights the importance of considering evaporation effects when conducting the viscosity measurement for molten zirconia. To assess the mass loss caused by evaporation, a series of evaporation tests were conducted. In these tests, zirconia samples were levitated and heated to form droplets, and the droplet temperatures were controlled at approximately 3200K for a certain duration. The sample mass was measured before and after the tests to quantify the mass loss resulting from evaporation. Considering the duration of sample levitation, the average evaporation rate of molten zirconia was determined to be approximately 8 μg/s for a sample with a mass of about 20 mg levitated at 3200K. This corresponds to a mass loss of approximately 12 % during a 5-min levitation period. As the temperature increases, the evaporation rate is expected to accelerate. Therefore, it is recommended to complete one round of viscosity measurement within approximately 2–3 min to minimize the impact of evaporation. The sample should be weighed before and after a levitation to accurately account for the mass loss due to evaporation and ensure the reliability of the viscosity data.

Molten zirconia has an extremely low viscosity, typically only a few mPa·s. This characteristic, combined with its high density of approximately 5000 kg/m^3^, allows molten zirconia to flow over long distances during core meltdown in light water nuclear reactors. The low viscosity of molten zirconia plays a vital role in facilitating the spreading and subsequent cooling of corium, making it a critical parameter for predicting the progression of severe accidents. However, obtaining accurate viscosity data for molten zirconia is challenging due to several factors. Firstly, the melting temperature of zirconia is significantly higher compared to alumina (2988K for molten zirconia and 2327K for molten alumina). Traditional contact methods face limitations in covering temperatures above 3000K. Furthermore, the higher evaporation rate of zirconia presents additional difficulties in viscosity measurements and necessitates shorter measurement times. In the present study, the evaporated zirconia tends to solidify on the low-temperature nozzle wall, leading to a reduction in the flatness of the nozzle surface. As a result, the levitation becomes unstable, and the measurement cannot continue. It is then necessary to clean the nozzle surface before resuming the viscosity measurement. Advanced experimental techniques and careful control over experimental conditions are still needed to overcome these difficulties and obtain more reliable viscosity data for molten zirconia, contributing to a better understanding of its flow behavior and its impact on reactor safety. When it comes to the viscosity measurement of materials containing uranium dioxide, The levitation stability will further deteriorate since uranium dioxide has a much higher evaporation rate than zirconia.

In future research, the measurement of thermophysical properties of corium components, such as UO_2_/ZrO_2_/Zr/Fe and their mixtures, holds significant importance. These measurements are essential for a comprehensive understanding of corium behavior, particularly in the context of nuclear reactor safety. Higher vapor pressure associated with UO_2_ samples requires careful handling and strict control over the gas environment to minimize the evaporation rate and protect researchers. Specialized techniques and methodologies need to be developed to ensure accurate measurements while maintaining safety. In the case of mixtures, the evaporation of volatile components can lead to changes in the composition of the sample. This aspect needs to be taken into account during the measurement process to ensure accurate and representative results. Ongoing advancements in experimental methodologies and techniques, coupled with the collective expertise of researchers in this field, will play a pivotal role in overcoming these challenges and obtaining valuable data on the thermophysical properties of corium components in the future.

## 4 Conclusions

This paper presents an experimental setup (ALSEE) developed at CNPE for accurate measurements of density, surface tension and viscosity of molten corium components using aerodynamic levitation, laser heating and droplet oscillation techniques. The obtained viscosity values of molten alumina and molten zirconia demonstrate the effectiveness of the ALSEE facility.

The viscosity of molten alumina was determined to be 47.06 ± 2.15 mPa s at its melting temperature of 2327K. The viscosity decreases as the temperature increases. The activation energy for molten alumina was determined to be (1.546 ± 0.144) × 10^5^ J/mol. For molten zirconia, the viscosity at its melting temperature of 2988K was found to be 12.87 ± 1.03 mPa s. Similar to alumina, the viscosity of molten zirconia decreases with increasing temperature. The activation energy for molten zirconia was determined to be (8.035 ± 2.010) × 10^4^ J/mol, with a relatively large uncertainty due to fluctuations in viscosity values.

The ALSEE facility offers a reliable and advanced approach to the thermophysical property measurements of corium components with the aim of enhancing our knowledge in understanding the corium behavior and its impact on reactor safety.

## Data availability statement

Research data has been deposited into a publicly available repository: Gong, Yaopeng (2023), “viscosity data of molten alumina and zirconia”, Mendeley Data, V4, https://doi.org/10.17632/yftyj2cj9f.4. Data can be found online at https://data.mendeley.com/datasets/yftyj2cj9f/4.

## CRediT authorship contribution statement

**Yaopeng Gong:** Conceptualization, Investigation, Methodology, Writing – original draft, Writing – review & editing. **Li zhang:** Conceptualization, Formal analysis, Investigation, Methodology. **Yidan Yuan:** Data curation, Funding acquisition, Project administration, Writing – review & editing. **Qiang Guo:** Formal analysis, Investigation, Methodology. **Weimin Ma:** Conceptualization, Methodology, Writing – original draft, Writing – review & editing. **Shanfang Huang:** Formal analysis, Methodology, Writing – original draft, Writing – review & editing.

## Declaration of competing interest

The authors declare that they have no known competing financial interests or personal relationships that could have appeared to influence the work reported in this paper.
